# Effects of SGLT2 inhibitors on NSAID-associated acute kidney injury in type 2 diabetes: a claims-based cohort study

**DOI:** 10.1186/s12882-026-04753-z

**Published:** 2026-01-16

**Authors:** Yuki Kunitsu, Hiroyoshi Koide, Keiko Ikuta, Daiki Hira, Shunsaku Nakagawa, Masahiro Tsuda, Shin-Ya Morita, Tomohiro Terada

**Affiliations:** 1 Department of Clinical Pharmacology and Therapeutics, Kyoto University Hospital, 54 Shogoin-Kawahara-cho, Sakyo-ku, Kyoto, 606-8507 Japan; 2 Department of Pharmacy, Shiga University of Medical Science Hospital, Seta Tsukinowa-cho, Otsu, Shiga 520-2192 Japan; 3 Graduate School of Pharmaceutical Sciences, Kyoto University, 46-29 Yoshida-Shimo-Adachi-cho, Sakyo-ku, Kyoto, 606-8501 Japan

## Abstract

**Background:**

Sodium–glucose cotransporter-2 inhibitors (SGLT2is) reduce the risk of acute kidney injury (AKI) in diverse populations; however, their effects on drug-induced AKI remain unclear. Nonsteroidal anti-inflammatory drugs (NSAIDs) are a major cause of nephrotoxicity, and combining them with renin–angiotensin–aldosterone system inhibitors and diuretics increases the risk. Given the natriuretic action of SGLT2is, their impact on NSAID-related AKI requires evaluation.

**Methods:**

We performed this retrospective cohort study using the nationwide Japanese claims database (2015–2023). Adults with type 2 diabetes initiated on NSAIDs while receiving either SGLT2is or dipeptidyl peptidase-4 inhibitors (DPP4is) were included. Inverse probability of treatment weighting balanced 60 covariates. The primary outcome was 90-day AKI, identified by International Classification of Diseases 10th edition codes, and the secondary outcomes were 30-day risk and on-treatment incidence during NSAID use.

**Results:**

We identified 39,251 SGLT2i and 256,298 DPP4i users. After weighting, the covariates were well balanced. The occurrence rates of 90-day AKI were 0.17% and 0.24% in SGLT2i and DPP4i users, respectively (risk ratio 0.70, 95% confidence interval: 0.62–0.79). Consistent protection was observed at 30 days and during on-treatment exposure. Subgroup analyses showed greater benefit in younger patients and those with chronic kidney disease, higher BMIs, and shorter exposure. None of the subgroups demonstrated excess risk.

**Conclusions:**

SGLT2i therapy was associated with a significantly lower risk of NSAID-induced AKI than DPP4is. These findings indicate an observed association between SGLT2i therapy and a lower incidence of drug-induced nephrotoxicity, which may inform safer NSAID prescribing in patients with type 2 diabetes.

**Clinical trial number:**

Not applicable.

**Supplementary information:**

The online version contains supplementary material available at 10.1186/s12882-026-04753-z.

## Key points

Keypoints Sodium–glucose cotransporter-2 inhibitors (SGLT2is) were associated with a significantly lower risk of acute kidney injury (AKI) after nonsteroidal anti-inflammatory drug (NSAID) use in patients with type 2 diabetes. This association was consistent across multiple analyses and patient subgroups, suggesting that SGLT2is may be linked to lower risk of drug-induced kidney injury when NSAIDs are clinically required. These findings extend t current knowledge of SGLT2i renal safety and provide real-world evidence of an association between SGLT2i use and reduced NSAID-related nephrotoxicity.

**Supplementary information:**

The online version contains supplementary material available at 10.1186/s12882-026-04753-z.

## Introduction

Sodium–glucose cotransporter-2 inhibitors (SGLT2is) were originally developed as glucose-lowering agents for type 2 diabetes mellitus (T2DM) and have since been approved for the treatment of chronic heart failure and chronic kidney disease (CKD). Large randomized trials and meta-analyses have demonstrated that SGLT2is slow the progression of renal dysfunction, delay the need for dialysis in patients with CKD, and reduce the risk of acute kidney injury (AKI) by approximately 23–25% in broad clinical populations [1–3]. Given their protective effect on the kidneys, SGLT2is play an important role in the treatment and prevention of CKD [4–6]. However, except for reports on some drugs, the effect of SGLT2is on drug-induced AKI remains poorly characterized [7]. Nonsteroidal anti-inflammatory drugs (NSAIDs) decrease renal perfusion and increase AKI risk, an effect that is exacerbated by concurrent diuretic or renin–angiotensin–aldosterone system inhibitor (RASI) therapy [8, 9]. Despite the natriuretic and diuretic properties of SGLT2is, the effects of these agents on NSAID-associated AKI have not been systematically studied. Thus, we conducted a retrospective claims-based analysis to assess whether SGLT2i use mitigates the risk of NSAID-induced AKI in patients with T2DM.

## Methods

### Data sources

This retrospective cohort study used data from the DeSC database provided by DeSC Healthcare, Inc. (Tokyo, Japan). The database contains medical claims data of approximately 12 million individuals across various Japanese insurance systems, with an age distribution representative of the Japanese population [10, 11].

### Study design

This retrospective, active-comparator cohort study included patients with T2DM initiated on NSAID therapy while receiving either an SGLT2i or a dipeptidyl peptidase-4 inhibitor (DPP4i) treatment from April 1, 2015, to March 31, 2023. Patients with T2DM were identified as those who had at least one recorded diagnosis with an ICD-10 code E10 or E14 (without the “suspected” flag) in the claims database within one year before the index date. We selected DPP4is as the comparator class because SGLT2is and DPP4is occupy similar positions in type 2 diabetes management in Japan [12] and because DPP4is exert minimal effects on renal outcomes [13]. Eligible patients were adults (≥20 years) with ≥365 days of continuous enrollment before NSAID initiation, no AKI or dialysis in the prior 365 days, no NSAID or comparator-drug use in the 90 days before the index date, and follow-up of at least 1 day after the day following NSAID initiation. The index date was defined as the date of first NSAID dispensation during SGLT2i or DPP4i therapy. For the primary analysis, we calculated the incidence of IPTW-adjusted AKI within 90 days of NSAID initiation. In secondary analyses, we similarly estimated the incidence of IPTW-adjusted AKI within 30 days of initiation and during NSAID co-administration (up to 90 days).

### Exposure

Target drugs (SGLT2i, DPP4i, and NSAIDs) were defined based on the anatomical therapeutic chemistry (ATC) classification system and are listed in Supplementary Table S1. Topical drugs such as tapes and ointments were excluded. The duration of use of each drug was defined as the period from the date of dispensing to the number of days for which the drug was prescribed, excluding as-needed prescriptions. Given that patient adherence to medications and follow-up consultations may not have been perfect, a 30-day grace period was used. In other words, a drug was considered to be in continuous use if the next prescription was dispensed within 30 days of the previous drug use plus the prescribed number of days.

### Definition of covariates and outcome

Propensity scores were estimated from 60 baseline characteristics selected to capture factors influencing the choice of SGLT2i versus DPP4i therapy and well-established predictors of AKI [14–24]. These included demographic and utilization factors (age, sex, calendar year, recent hospitalizations, and surgical procedures), key clinical comorbidities (such as diabetic complications, hypertension, heart failure, and CKD), background glucose-lowering therapies (for example, insulin, metformin, and sulfonylureas) along with selected concomitant medications (including RASIs, diuretics, and antibiotics), and health-check parameters (receipt of a check within the prior year and, where available, body mass index (BMI), hemoglobin A1c (HbA1c), blood pressure, proteinuria grade, and estimated glomerular filtration rate (eGFR)). Detailed definitions and code lists are provided in Supplementary Tables S2–S4. Calendar year of the index date was included as an ordinal covariate in the propensity score model to adjust for temporal trends in patient characteristics and prescribing practices. Urine glucose was excluded from propensity score estimation because its positivity directly reflects the pharmacological action of SGLT2i. The primary outcome of this study was the new diagnosis of AKI, identified using the International Classification of Diseases, 10th edition (ICD-10) code N17X. Previous studies have indicated that the ICD-10 code N17X has moderate sensitivity and high specificity for identifying AKI [25]. For dialysis, the date of the act with Japanese procedure code J038 or C102 was defined as the date of dialysis. Surgical procedures were defined using Japanese procedure codes K00–91 and K93.

### Statistical analysis

Propensity scores were estimated from the 60 baseline characteristics described above via multivariable logistic regression, and inverse probability of treatment weighting (IPTW) was applied to balance the covariates between the SGLT2i and DPP4i cohorts. To avoid the undue influence of extreme weights, any IPTW values below the 1st percentile or above the 99th percentile were truncated to the percentile thresholds. We assessed the covariate balance after IPTW by calculating the standardized mean differences (SMDs) for each baseline characteristic. Although an SMD < 0.10 is ideal, SMDs < 0.25 are generally considered acceptable [26].

In the primary analysis, we used IPTW-adjusted weights to calculate the proportion of patients who experienced AKI within 90 days of NSAID initiation and then derived the risk ratio by comparing SGLT2i versus DPP4i. As secondary analyses, we (1) calculated the IPTW-adjusted AKI proportion within 30 days post-initiation and derived the corresponding 30-day risk ratio, and (2) estimated the on-treatment AKI incidence during the actual NSAID co-administration period (capped at 90 days) per 100 person-years using the Poisson method, from which we obtained the incidence rate ratio. For the on-treatment analysis, follow-up was censored at the earliest AKI occurrence, NSAID discontinuation, SGLT2i or DPP4i discontinuation, addition of the comparator drug class, loss of enrollment, or 90 days after the index date. All hypothesis tests were two-sided with α = 0.05. Confidence intervals for on-treatment incidence rates were computed in Python 3.11 using the Poisson distribution; all other analyses were performed in JMP® Pro 18 (SAS Institute, Cary, NC, USA).

### Subgroup analyses

To explore effect modification, we conducted 13 predefined subgroup analyses. Within each subgroup, patients were re-stratified, and propensity scores were re-estimated using the same 60 covariates as in the primary analysis, followed by IPTW to balance baseline characteristics. The incidence of AKI within each subgroup was compared between the SGLT2i and DPP4i cohorts using IPTW-adjusted proportions, and the corresponding risk ratios were derived. The subgroups were as follows:

1. Age ≥ 75 versus < 75 years
2. History of CKD versus no CKD
3. History of chronic heart failure versus none.
4. Presence versus absence of diabetic complications
5. Concomitant RASI use versus none
6. Concomitant diuretic use versus none
7. Combined both RASI and diuretic use versus none
8. BMI ≥ 25 kg/m^2^ versus < 25 kg/m^2^
9. eGFR ≥ 60 mL/min/1.73 m^2^ versus < 60 mL/min/1.73 m^2^
10. Continuation of SGLT2i/DPP4i for ≥ 90 versus < 90 days
11. NSAID co-initiation in summer (June–September) versus other months
12. Received parameters of specific health checkups within the prior year versus none
13. Calendar year on index date (2015–2019 versus 2020–2023)

Among these, the age cutoff of 75 years was selected because it corresponds to the threshold for the medical care system for elderly in the latter stage of life in Japan, at which point the health insurance system and medical service structure differ from those for younger adults. In addition, the calendar year subgroup (2015–2019 vs. 2020–2023) was defined to correspond to the periods before and after the expansion of SGLT2i indications for heart failure and CKD in Japan, which may have influenced prescribing patterns and patient characteristics.

### Sensitivity analyses

To evaluate the robustness of our findings, we conducted two sensitivity analyses:

1. Propensity score matching (PSM): We performed 1:1 nearest neighbor matching on the logit of the propensity score using a caliper width of 0.2 standard deviations. After matching, the covariate balance was confirmed using standardized mean differences, and AKI incidence was compared between the matched SGLT2i and DPP4i cohorts using proportions in the matched cohort and the corresponding risk ratios.
2. Expanded outcome definition: We repeated the primary IPTW analysis using an expanded AKI definition: any patient with an ICD-10 code for acute kidney injury (N17X), acute tubulointerstitial nephritis (N10), or with initiation of dialysis within 90 days of NSAID initiation was counted as an AKI event, while all other analytical steps (weighting, censoring rules, and risk-ratio calculation) remained identical to the main analysis.

### Ethics

The study was conducted in accordance with the principles of the Declaration of Helsinki. According to the Japanese Ethical Guidelines for Medical and Biological Research Involving Human Subjects, the use of fully anonymized secondary data is exempt from institutional review board approval and informed consent. Therefore, informed consent and institutional review board approval were not required for this study.

## Results

**Fig. 1:**
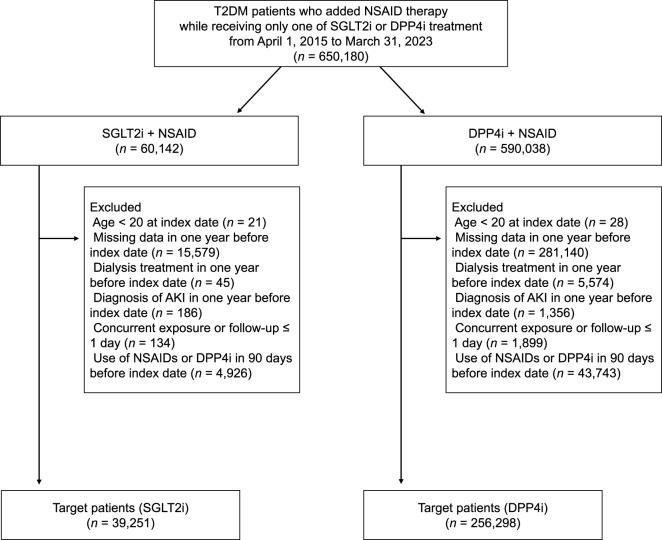
Flowchart of target patient inclusion. The index date was defined as the date of first NSAID dispensation during SGLT2i or DPP4i therapy. AKI, acute kidney injury; DPP4i, dipeptidyl peptidase-4 inhibitor; NSAID, nonsteroidal anti-inflammatory drug; SGLT2i, sodium-glucose cotransporter-2 inhibitor; T2DM, type 2 diabetes mellitus

The final analytical cohorts comprised 39,251 SGLT2i and 256,298 DPP4i users (Fig. 1). The baseline characteristics of the cohorts before and after IPTW are shown in Supplementary Table S5. Before weighting, several key covariates, such as age, calendar year, CKD history, heart failure history, and concomitant diuretic use, exhibited an imbalance. After applying IPTW with weights truncated at the 1st and 99th percentiles, all 60 covariates attained an acceptable balance (post-IPTW SMDs < 0.25, ideal < 0.10 for most variables), including age (SMD = 0.14), confirming successful adjustment. The primary and secondary outcomes are summarized in Table 1. In the 90-day primary analysis, the IPTW-weighted AKI incidence was 0.17% in the SGLT2i cohort versus 0.24% in the DPP4i cohort, corresponding to a risk ratio (RR) of 0.70 [95% confidence interval (CI): 0.62–0.79]. In secondary analyses, the 30-day AKI incidence was 0.14% versus 0.21% (RR 0.66 [95% CI: 0.58–0.76]), and the on-treatment AKI rate during NSAID co-administration was 12.4 versus 16.6 events/1,000 person-years (incidence rate ratio 0.75 [95% CI: 0.62–0.90]).

**Table 1:** Primary and secondary outcomes

Outcomes	Before IPTW						After IPTW					
	SGLT2i patients (*n* = 39,251)		DPP4i patients (*n* = 256,298)		RR or IRR [95% CI]		SGLT2i patients (*n* = 256,983)		DPP4i patients (*n* = 295,853)		RR or IRR [95% CI]	
AKI within 90 days of NSAID initiation, n, (%)	72	(0.18)	571	(0.22)	0.82	[0.64–1.05]	432	(0.17)	710	(0.24)	0.70	[0.62–0.79]
AKI within 30 days of NSAID initiation, n, (%)	59	(0.15)	489	(0.19)	0.79	[0.60–1.03]	356	(0.14)	618	(0.21)	0.66	[0.58–0.76]
AKI incidence during NSAID co-administration (up to 90 days), n, (%)	29	(0.07)	225	(0.09)	−		169	(0.07)	281	(0.09)	−	
Duration during NSAID co-administration (up to 90 days), days	745,425		5,335,883		−		4,970,064		6,166,718		−	
AKI incidence during NSAID co-administration (up to 90 days), 1000 person-years, [95% CI]	14.2	[9.30−19.6]	15.4	[13.4−17.4]	0.92	[0.63–1.36]	12.4	[10.6−14.3]	16.6	[14.7−18.6]	0.75	[0.62–0.90]

After IPTW: the displayed “n” represents the sum of IPTW weights

AKI, acute kidney injury. CI, confidence interval. IRR: Incidence rateratio. NSAID, nonsteroidal anti-inflammatory drugs. RR, risk ratio

### Subgroup analysis

In the subgroup analyses (Table 2), the AKI risk reduction with SGLT2i versus DPP4i was particularly pronounced among patients aged < 75 years (RR 0.44 [95% CI: 0.34–0.59]), those with CKD history (RR 0.51 [95% CI: 0.38–0.68]), those who received parameters of specific health checkups within the prior year (RR 0.51 [95% CI: 0.34–0.77]), those with BMI ≥ 25 kg/m^2^ (RR 0.26 [95% CI: 0.11–0.60]), and those whose SGLT2i/DPP4i exposure prior to NSAID initiation was < 90 days (RR 0.24 [95% CI: 0.16–0.36]). Although several subgroups (e.g., RASI co-users and heart failure-free patients) did not reach statistical significance, no subgroup demonstrated a higher risk of AKI with SGLT2i.

**Table 2:** Primary outcomes in the subgroups

Subgroups	AKI within 90 days of NSAID initiation after IPTW					
	SGLT2i patients, weighted n, (%)		DPP4i patients, weighted n, (%)		RR [95% CI]	
1. Age						
≥75 years	351	(0.24)	545	(0.30)	0.81	[0.71–0.93]
< 75 years	69	(0.06)	160	(0.14)	0.44	[0.34–0.59]
2. CKD history						
History of CKD	68	(0.34)	152	(0.67)	0.51	[0.38–0.68]
No history of CKD	364	(0.15)	560	(0.21)	0.75	[0.66–0.86]
3. Heart failure history						
History of heart failure	162	(0.21)	361	(0.41)	0.50	[0.41–0.60]
No history of heart failure	272	(0.15)	340	(0.16)	0.94	[0.80–1.10]
4. Diabetic complications						
Presence of diabetic complications	221	(0.21)	322	(0.26)	0.80	[0.68–0.95]
Absence of diabetic complications	225	(0.15)	377	(0.22)	0.68	[0.58–0.80]
5. Concomitant RASI use						
Concomitant RASI use	314	(0.24)	391	(0.27)	0.91	[0.78–1.05]
No concomitant RASI use	118	(0.09)	319	(0.21)	0.43	[0.35–0.54]
6. Concomitant diuretic use						
Concomitant diuretic use	191	(0.44)	287	(0.59)	0.74	[0.62–0.89]
No concomitant diuretic use	237	(0.11)	410	(0.17)	0.67	[0.57–0.79]
7. Combined both RASI and diuretic use						
Combined both RASI and diuretic use	138	(0.46)	167	(0.51)	0.90	[0.72–1.13]
Combined neither of RASI and diuretic use	61	(0.05)	194	(0.14)	0.37	[0.28–0.49]
8. BMI						
BMI ≥ 25 kg/m^2^	7	(0.03)	31	(0.11)	0.26	[0.11–0.60]
BMI < 25 kg/m^2^	24	(0.10)	48	(0.12)	0.82	[0.50–1.34]
9. eGFR						
eGFR ≥ 60 mL/min/1.73 m^2^	0	(0)	22	(0.06)	N/A	
eGFR < 60 mL/min/1.73 m^2^	27	(0.20)	48	(0.31)	0.65	[0.41–1.04]
10. Continuation of SGLT2i/DPP-4i						
Continuation of SGLT2i/DPP-4i for ≥ 90 days	415	(0.19)	573	(0.22)	0.85	[0.75–0.96]
Continuation of SGLT2i/DPP-4i for < 90 days	28	(0.08)	126	(0.32)	0.24	[0.16–0.36]
11. Season of NSAID co-initiation						
Summer (June–September)	142	(0.17)	238	(0.23)	0.72	[0.58–0.88]
Other months	285	(0.17)	473	(0.24)	0.69	[0.60–0.80]
12. Received parameters of specific health checkups within the prior year						
Received parameters of specific health checkups within the prior year	33	(0.06)	72	(0.13)	0.51	[0.34–0.77]
Not received parameters of specific health checkups within the prior year	402	(0.20)	639	(0.27)	0.73	[0.65–0.83]
13. Calendar year on index date						
2015−2019	61	(0.07)	249	(0.21)	0.33	[0.25–0.44]
2020−2023	361	(0.21)	472	(0.27)	0.81	[0.70–0.93]

AKI, acute kidney injury; BMI, body mass index; CKD, chronic kidney disease; DPP4i, dipeptidyl peptidase-4 inhibitor; IPTW, inverse probability of treatment weighting; N/A, not applicable; NSAID, nonsteroidal anti-inflammatory drug; eGFR, estimated glomerular filtration rate; RASI, renin–angiotensin–aldosterone system inhibitor; RR, risk ratio; SGLT2i, sodium–glucose cotransporter-2 inhibitor

### Sensitivity analysis

#### Propensity score matching

In the propensity score-matched cohort (*n* = 33,839 pairs; balance shown in Supplementary Table S6), the PSM-based 90-day AKI incidence remained significantly lower in the SGLT2i group than in the DPP4i group (0.17% vs. 0.26%; RR 0.67 [95% CI: 0.48–0.93]), and the 30-day incidence likewise favored SGLT2i (0.14% vs. 0.22%; RR 0.64 [95% CI: 0.45–0.92]) (Supplementary Table S7). However, when considering the on-treatment AKI incidence (events per 1000 person-years during NSAID co-administration), the difference was no longer statistically significant (12.4 vs. 17.7; incidence rate ratio 0.70 [0.41–1.20]).

#### Expanded outcome definition

When the expanded AKI definitions were applied as sensitivity checks, the protective effect of SGLT2i remained consistent. First, defining AKI as either ICD-10 code N17X or N10 diagnosis within 90 days yielded an IPTW-adjusted RR of 0.70 [95% CI: 0.65–0.76] (Supplementary Table S8). Second, defining AKI as either ICD-10 code N17X diagnosis or initiation of dialysis within 90 days produced an IPTW-adjusted RR of 0.65 [95% CI: 0.58–0.72].

## Discussion

In this large, retrospective cohort study of 39,251 SGLT2i and 256,298 DPP4i users who initiated NSAID therapy, SGLT2i co-administration was associated with a 30% lower 90-day risk of AKI (RR 0.70 [95% CI: 0.62–0.79]). This association remained consistent in the secondary analyses of 30-day AKI risk and showed a similar trend during actual NSAID exposure. No subgroup experienced an increased AKI risk with SGLT2i, and these stronger relative associations, compared with the overall cohort, were observed in patients aged < 75 years, those with a history of heart failure, those without concomitant RASI use, those without concomitant RASI and diuretic use, those with BMI ≥25 kg/m^2^, and those with < 90 days of prior SGLT2i/DPP-4i exposure. A similar trend toward greater AKI risk reduction was observed among patients with a history of CKD. The subgroup analysis by calendar year revealed that the relative risk reduction of NSAID-associated AKI with SGLT2i use was greater in the earlier period (2015–2019) than in the later period (2020–2023). The observed temporal heterogeneity in effect estimates likely reflects changes in the clinical characteristics of treated patients and prescribing patterns following the expansion of SGLT2i indications in Japan. After 2020, with the expansion of SGLT2i indications to heart failure and CKD in Japan, the treated population likely became older and included more patients with reduced eGFR and multiple comorbidities, while the characteristics of DPP4i users remained relatively stable. Consequently, the relative difference in AKI risk between SGLT2i and DPP4i users narrowed. However, the direction of the association between SGLT2i use and lower AKI risk remained consistent across periods and was also observed within high-risk subgroups such as those with CKD or heart failure.

Our findings provide evidence for the protective AKI signal of SGLT2i observed in randomized trials in diabetes and CKD populations [1–3] in a real-world, NSAID-exposed cohort. Previous observational studies have reported lower AKI rates in SGLT2i users than in DPP4i users [27, 28] or SGLT2i non-users [29]; our active-comparator design and robust IPTW adjustment indicate that this association persists even in the context of NSAID-induced AKI. Notably, a pharmacovigilance and claims-database study using the Food and Drug Administration adverse event reporting system and the Japanese hospital-based claims database likewise found that SGLT2i users had significantly lower odds of NSAID-associated AKI versus DPP4i users (reporting odds ratio 0.65 [95% CI: 0.48–0.88]; odds ratio 0.46 [0.41–0.53]) [30]. However, the effects of comorbidities and concomitant medications were not assessed. Numerous observational studies have compared SGLT2is with DPP-4is regarding cardiovascular and renal outcomes [27, 28, 31]. By comparing against a renally neutral agent, we minimize bias from differential prescribing patterns or diabetes severity and strengthen the inference that the observed AKI risk reduction is attributable to the unique renal effects of SGLT2i rather than the actions of comparator drugs.

SGLT2is protect the kidneys through multiple, interrelated mechanisms, chiefly by restoring tubuloglomerular feedback (TGF) [32–36], inducing osmotic diuresis and natriuresis [37, 38], modulating sympathetic activity [39, 40] and aldosterone signaling [41], and exerting anti-inflammatory and antifibrotic effects [42]. Other mechanisms have also been proposed [43–45]. Inhibiting proximal sodium–glucose reabsorption increases distal tubular sodium delivery, re-engages TGF to normalize afferent arteriolar tone, and lowers intraglomerular pressure [32–35]. Meanwhile, NSAIDs induce afferent arteriolar vasoconstriction by inhibiting prostaglandin synthesis [46]. Therefore, the restoration of TGF is hypothesized to directly counteract NSAID-mediated afferent constriction. Concurrent natriuresis and modest diuresis reduce interstitial edema [47] and support medullary oxygenation [48]. Previous studies have also demonstrated tubular epithelial preservation via anti-inflammatory and antifibrotic pathways [42]. In our NSAID-treated cohort, these hemodynamic and cellular effects manifested as a significant AKI risk reduction. Subgroup findings reinforce this mechanism: patients with BMI ≥25 kg/m^2^ who often exhibit systemic glomerular hyperfiltration and patients with CKD whose remaining nephrons compensate via hyperfiltration due to nephron loss showed the greatest relative benefit, which may reflect their larger “reserve” for TGF-mediated pressure normalization. Notably, even short-term SGLT2i exposure ( < 90 days) conferred protection, indicating that TGF restoration occurred rapidly. Dapagliflozin markedly increased early distal tubular chloride delivery by 70% in diabetic rats, immediately re-engaging TGF to normalize afferent arteriolar tone [34]. Studies in humans similarly demonstrated that a single SGLT2i dose rapidly restores TGF responsiveness, as reflected by prompt stabilization of intraglomerular hemodynamics well before long-term renal benefits emerge [36]. Younger patients ( < 75 years) derived more benefits, likely due to preserved nephron TGF responsiveness and tubular reserve. A previous study suggested that combining SGLT2i with RASI could further reduce fibrosis, inflammation, and glomerular injury in diabetic nephropathy [49]. However, in our NSAID-induced AKI setting, the incremental benefits were attenuated in patients receiving RASI. Furthermore, studies have shown that angiotensin II can enhance NaCl-triggered TGF responses via the activation of angiotensin II subtype 1 receptors on macula densa cells [50]. RASI already dilate the efferent arteriole and lower intraglomerular pressure [51, 52], partly overlapping with the hemodynamic actions of SGLT2i. Therefore, this attenuation may reflect the overlapping effects on glomerular hemodynamics rather than a lack of additive renal protection. Similarly, among patients without heart failure, incremental benefits were attenuated. Baseline glomerular hypertension and neurohormonal activation [53] are less pronounced in this group. Hence, the “excess” afferent arteriolar dilation available for correction by SGLT2i-mediated TGF reactivation may be smaller, resulting in a comparatively reduced AKI risk reduction. Collectively, these data support a model in which SGLT2 inhibitors guard against NSAID-induced AKI, an injury driven primarily by afferent arteriole constriction leading to renal ischemia [54], by reestablishing TGF, thereby bolstering renal hemodynamic stability.

This study has several limitations that warrant consideration. First, although the DeSC database used in this study includes multiple types of public insurance and is broadly representative of the Japanese population, it does not encompass all insurance types or regions. Therefore, caution is warranted when generalizing these findings to populations not represented in this database. This analysis also relied on administrative claims data supplemented by health-check information for only a subset of patients. Thus, we could not obtain serial laboratory measures of renal function for everyone; accordingly, AKI was defined using ICD-10 codes (N17/N10), which—while shown to have moderate sensitivity and high specificity [25]—may not capture all subclinical or transient events. Second, unmeasured factors, such as individual hydration status, over-the-counter NSAID use, and medication adherence, were unavailable in our dataset and could have influenced the risk of AKI. Third, as with any observational study, residual confounding remains possible despite extensive covariate adjustments. Although the inverse probability of treatment weighting and propensity score matching achieved robust covariate balance, and multiple sensitivity analyses (including expanded AKI definitions and matched comparisons) yielded consistent results, the E-value for the observed risk ratio (0.70) was 2.21 (and 1.85 for the upper limit of the 95% confidence interval) [55, 56], indicating that a relatively strong unmeasured confounder would be required to fully explain the observed association. Finally, although the relative association between SGLT2i use and lower AKI risk was statistically significant, the clinical relevance of the observed absolute risk reduction of 0.07% remains to be determined. The diagnosis of AKI in this study relied on ICD-10 codes, which are known to have high specificity but limited sensitivity [25]; therefore, the true incidence and absolute risk reduction might be underestimated. Future studies incorporating laboratory data are warranted to clarify the absolute magnitude and clinical impact of this association. Nevertheless, the possibility of residual confounding cannot be completely excluded, and the findings should be interpreted as an association between SGLT2i use and a lower incidence of NSAID-associated AKI rather than a definitive causal relationship.

For clinicians managing patients with T2DM requiring NSAIDs, these findings suggest that SGLT2i therapy may be associated with a lower risk of AKI compared with DPP4i therapy. While our results add to the growing body of real-world evidence supporting the renal safety profile of SGLT2is, prospective studies and mechanistic investigations are warranted to confirm these observations and clarify their clinical implications.

## Electronic supplementary material

Below is the link to the electronic supplementary material.

Supplementary Material 1

## Data Availability

The data that support the findings of this study are available from DeSC Healthcare Inc., but restrictions apply to the availability of these data, which were used under the license for the current study and are not publicly available. However, the data are available from the authors upon reasonable request and with permission from DeSC Healthcare Inc.

## Abbreviations

AKI: Acute kidney injury
ATC: Anatomical therapeutic chemistry
BMI: Body mass index
CKD: Chronic kidney disease
CI: Confidence interval
DPP4i: Dipeptidyl peptidase-4 inhibitor
eGFR: Estimated glomerular filtration rate
HbA1c: Hemoglobin A1c
ICD-10: International Classification of Diseases, 10th edition
IPTW: Inverse probability of treatment weighting
NSAIDs: Nonsteroidal anti-inflammatory drugs
PSM: Propensity score matching
RASI: Renin–angiotensin system inhibitor
RR: Risk ratio
SGLT2i: Sodium–glucose cotransporter-2 inhibitor
SMD: Standardized mean difference
T2DM: Type 2 diabetes mellitus
TGF: Tubuloglomerular feedback
