# Supplementary material for: Effects of SGLT2 inhibitors on NSAID-associated acute kidney injury in type 2 diabetes: a claims-based cohort study
Source: BMC Nephrol. 2026 Jan 16;27:106. doi: 10.1186/s12882-026-04753-z (PMC12892778; doi:10.1186/s12882-026-04753-z)
Supplement: Supplementary file 1 — Supplementary Material 1 [file 12882_2026_4753_MOESM1_ESM.pdf]

Supplementary Materials

Effects of SGLT2 Inhibitors on NSAID-Associated Acute Kidney Injury in Type 2 Diabetes: A Claims-Based Cohort Study

BMC Nephrology

Authors: Yuki Kunitsu, Hiroyoshi Koide, Keiko Ikuta, Daiki Hira, Shunsaku Nakagawa, Masahiro Tsuda, Shin-ya Morita, Tomohiro Terada

Corresponding author: Yuki Kunitsu
Department of Clinical Pharmacology and Therapeutics, Kyoto University Hospital, 54 Shogoin-Kawahara-cho, Sakyo-ku, Kyoto, 606-8507, Japan
Email: ykunitsu@kuhp.kyoto-u.ac.jp

Supplementary Table S1 Definition of SGLT2i, DPP4i, and NSAIDs
Supplementary Table S2 Definition of diseases based on the International Classification of Diseases, 10th Edition (ICD-10) diagnosis codes
Supplementary Table S3 Definitions of drugs based on ATC codes
Supplementary Table S4 Parameters used for specific health checkups and categorization
Supplementary Table S5 Baseline characteristics of the SGLT2i and DPP4i cohorts before and after IPTW
Supplementary Table S6 Baseline characteristics of the SGLT2i and DPP4i cohorts after PSM
Supplementary Table S7 Primary and secondary outcomes after PSM
Supplementary Table S8 Primary and secondary outcomes for the expanded outcome definition after IPTW

Supplementary Table S1 Definition of SGLT2i, DPP4i, and NSAIDs

Category	Based ATC codes	Drug name
SGLT2i	A10BK	Canagliflozin, Dapagliflozin, Empagliflozin, Ipragliflozin, Luseogliflozin, and Tofogliflozin
DPP4i	A10BH	Alogliptin, Anagliptin, Linagliptin, Omarigliptin, Saxagliptin, Sitagliptin, Teneligliptin, Trelagliptin, and Vildagliptin
NSAID	M01A	Acemetacin, Aluminum flufenamate, Ampiroxicam, Celecoxib, Diclofenac sodium, Etodolac, Flurbiprofen, Flurbiprofen axetil, Ibuprofen, Indomethacin, Indomethacin farnesyl, Ketoprofen, Lornoxicam, Loxoprofen sodium hydrate, Mefenamic acid, Meloxicam, Moffezolac, Nabumetone, Naproxen, Oxaprozin, Piroxicam, Planoprofen, Proglumetacin maleate, Surindac, Thiaprofenic acid, and Zartoprofen

Compounds containing these ingredients were also analyzed.

ATC, anatomical therapeutic chemistry; DPP4i, dipeptidyl peptidase-4 inhibitor; NSAID, nonsteroidal anti-inflammatory drug; SGLT2i, sodium–glucose cotransporter-2 inhibitor.

Supplementary Table S2 Definition of diseases based on the International Classification of Diseases, 10th Edition (ICD-10) diagnosis codes

Diseases	ICD-10 codes
AKI	N17
Angina pectoris	I20
Atrial fibrillation	I48
Cancer	C00-C97
CKD	N18
Dehydration	E86
Dyslipidemia	E78
Heart failure	I110 and I50
Hypertension	I10-I15
Liver diseases	B18-B19 and K70-K77
Myocardial infarction	I21-I24
Non-diabetic acidosis	E872
Obesity	E66
Other renal disease	N00-N16, N19, and N25-N29
Peripheral vascular disease	I70-I74
Rheumatoid arthritis	M5-M6
Stroke	I60-I64
T2DM	E11, E14
T2DM with ketoacidosis	E111 and E141
T2DM with neurological complications	E114 and E144
T2DM with ophthalmic complications	E113 and E143
T2DM with peripheral circulatory complications	E115 and E145
T2DM with renal complications	E112 and E142
Venous thromboembolism	I26, I80-I81, I820, and I822-I829

AKI, acute kidney injury; CKD, chronic kidney disease; T2DM, type 2 diabetes mellitus

Supplementary Table S3 Definitions of drugs based on ATC codes

Class name	ATC codes
Alpha glucosidase inhibitors	A10BF
Antibacterials	J01
Anticoagulant agents	B01AA, B01AE, B01AF, and B01AX
Antivirals	J05
Beta blocking agents	C07
Biguanides	A10BA
Corticosteroids	H02A
Calcium channel blockers with mainly vascular effects	C08C
Glinides	A10BX02, A10BX03, and A10BX08
Glucagon-like peptide-1 analogs	A10BJ
Histamine H2 receptor antagonists	A02BA
HMG-CoA reductase inhibitors	C10AA
Imeglimin	A10BX15
Insulins and analogs	A10A
Loop diuretics	C03CA
Opioids	N02A
Other lipid modifying agents	C10AB–C10AX
Platelet aggregation inhibitors	B01AC
Potassium-sparing agents	C03D
Proton pump inhibitors	A02BC
RASIs	C09
Sulfonylureas	A10BB
Thiazide-like diuretics	C03BA
Thiazides	C03AA
Thiazolidinediones	A10BG
Vasopressin antagonists	C03XA
X-ray contrast media, Iodinated	V08A

Compounds containing these ingredients were also analyzed.

ATC, anatomical therapeutic chemistry; RASIs, renin–angiotensin system inhibitors.

Supplementary Table S4 Parameters used for specific health checkups and categorization

Parameters	Categorization
BMI (kg/m²)	≥30, <30 and ≥25, <25 and ≥18.5, or <18.5
HbA1c (%)	≥8.0, <8.0 and ≥7.0, <7.0 and ≥6.0, or <6.0
Systolic blood pressure (mmHg)	≥140 or <140
Diastolic blood pressure (mmHg)	≥90 or <90
Urine glucose (Qualitative test)	-, ±, 1+, 2+, or 3+
Urine protein (Qualitative test)	-, ±, 1+, 2+, or 3+
eGFR (mL/min/1.73 m^2)	≥60, <60 and ≥30, or <30

BMI, body mass index; eGFR, estimated glomerular filtration rate; HbA1c, hemoglobin A1c.

Supplementary Table S5 Baseline characteristics of the SGLT2i and DPP4i cohorts before and after IPTW

Characteristics	Before IPTW					After IPTW				
	SGLT2i patients (n=39,251)		DPP4i patients (n=256,298)		SMD	SGLT2i patients (weighted n=256,983)		DPP4i patients (weighted n=295,853)		SMD
Male, n (%)	22,865	(58.3)	136,952	(53.4)	0.10	141,515	(55.1)	160,012	(54.1)	0.02
Age, years, average (SD)	70.8	(12.91)	75.7	(9.88)	0.47	73.5	(10.9)	75.0	(10.5)	0.14
Calendar year on index date, n (%)										
2015	39	(0.1)	2,532	(1.0)	0.12	571	(0.2)	2,573	(0.9)	0.09
2016	603	(1.5)	10,003	(3.9)	0.15	7,443	(2.9)	10,619	(3.6)	0.04
2017	1,459	(3.7)	24,109	(9.4)	0.23	16,941	(6.6)	25,581	(8.6)	0.08
2018	2,450	(6.2)	25,812	(10.1)	0.14	23,036	(9.0)	28,275	(9.6)	0.02
2019	4,491	(11.4)	46,274	(18.1)	0.19	41,279	(16.1)	50,712	(17.1)	0.03
2020	5,540	(14.1)	45,206	(17.6)	0.10	45,721	(17.8)	50,747	(17.2)	0.02
2021	8,339	(21.2)	46,780	(18.3)	0.08	52,464	(20.4)	55,194	(18.7)	0.04
2022	12,698	(32.4)	41,749	(16.3)	0.38	52,225	(20.3)	54,575	(18.4)	0.05
2023	3,632	(9.3)	13,772	(5.4)	0.15	17,305	(6.7)	17,518	(5.9)	0.03
Hospitalization on index date, n (%)	3,407	(8.7)	28,700	(11.2)	0.08	25,688	(10.0)	32,516	(11.0)	0.03
Surgical procedure within 30 days before index date, n (%)	4,599	(11.7)	34,172	(13.3)	0.05	32,700	(12.7)	39,056	(13.2)	0.01
Medical history, n (%)										
AKI	253	(0.6)	952	(0.4)	0.04	1,073	(0.4)	1,183	(0.4)	<0.01
Angina pectoris	12,922	(32.9)	62,236	(24.3)	0.19	66,684	(25.9)	75,262	(25.4)	0.01

Characteristics	Before IPTW			After IPTW		
	SGLT2i patients (n=39,251)	DPP4i patients (n=256,298)	SMD	SGLT2i patients (weighted n=256,983)	DPP4i patients (weighted n=295,853)	SMD
Atrial fibrillation	6,613 (16.8)	22,484 (8.8)	0.24	25,477 (9.9)	29,150 (9.9)	<0.01
Cancer	7,159 (18.2)	52,696 (20.6)	0.06	50,284 (19.6)	60,108 (20.3)	0.02
CKD	5,023 (12.8)	17,668 (6.9)	0.20	19,603 (7.6)	23,060 (7.8)	0.01
Dehydration	8,031 (20.5)	44,846 (17.5)	0.08	45,481 (17.7)	53,485 (18.1)	0.01
Dyslipidemia	31,476 (80.2)	188,033 (73.4)	0.16	195,968 (76.3)	219,801 (74.3)	0.05
Heart failure	15,861 (40.4)	71,584 (27.9)	0.27	76,667 (29.8)	87,763 (29.7)	<0.01
Hypertension	32,419 (82.6)	202,714 (79.1)	0.09	205,529 (80.0)	235,384 (79.6)	0.01
Liver diseases	15,579 (39.7)	84,068 (32.8)	0.14	91,725 (35.7)	100,035 (33.8)	0.04
Myocardial infarction	2,647 (6.7)	8,604 (3.4)	0.16	10,318 (4.0)	11,227 (3.8)	0.01
Non-diabetic acidosis	544 (1.4)	2,343 (0.9)	0.04	2,685 (1.0)	2,968 (1.0)	<0.01
Obesity	1,521 (3.9)	2,542 (1.0)	0.19	4,378 (1.7)	4,217 (1.4)	0.02
Other renal disease	7,894 (20.1)	42,168 (16.5)	0.10	43,418 (16.9)	50,404 (17.0)	<0.01
Peripheral vascular disease	10,890 (27.7)	59,391 (23.2)	0.11	61,668 (24.0)	70,342 (23.8)	0.01
Rheumatoid arthritis	1,696 (4.3)	9,897 (3.9)	0.02	10,237 (4.0)	11,621 (3.9)	<0.01
Stroke	6,078 (15.5)	46,577 (18.2)	0.07	42,655 (16.6)	52,837 (17.9)	0.03
T2DM with ketoacidosis	236 (0.6)	787 (0.3)	0.04	998 (0.4)	1,068 (0.4)	<0.01
T2DM with neurological complications	3,337 (8.5)	19,282 (7.5)	0.04	20,426 (7.9)	22,648 (7.7)	0.01
T2DM with ophthalmic complications	11,090 (28.3)	74,082 (28.9)	0.01	75,239 (29.3)	85,095 (28.8)	0.01
T2DM with peripheral circulatory complications	409 (1.0)	2,224 (0.9)	0.02	2,349 (0.9)	2,619 (0.9)	<0.01

Characteristics	Before IPTW			After IPTW		
	SGLT2i patients (n=39,251)	DPP4i patients (n=256,298)	SMD	SGLT2i patients (weighted n=256,983)	DPP4i patients (weighted n=295,853)	SMD
T2DM with renal complications	7,973 (20.3)	44,731 (17.5)	0.07	47,417 (18.5)	52,700 (17.8)	0.02
Venous thromboembolism	2,304 (5.9)	9,849 (3.8)	0.09	11,102 (4.3)	12,484 (4.2)	0.01
Medication, n (%)						
Drugs for diabetes mellitus						
Alpha glucosidase inhibitors	3,544 (9.0)	36,054 (14.1)	0.16	32,205 (12.5)	39,527 (13.4)	0.03
Biguanides	10,961 (27.9)	78,856 (30.8)	0.06	80,402 (31.3)	89,446 (30.2)	0.02
Glinides	2,106 (5.4)	17,183 (6.7)	0.06	16,627 (6.5)	19,198 (6.5)	<0.01
Glucagon-like peptide-1 analogs[a]	4,433 (11.3)	456 (0.2)	0.49	4,913 (1.9)	4,888 (1.7)	0.02
Imeglimin	75 (0.2)	145 (0.1)	0.04	257 (0.1)	196 (0.1)	0.01
Insulins and analogs	5,163 (13.2)	22,192 (8.7)	0.15	26,568 (10.3)	27,629 (9.3)	0.03
Sulfonylureas	3,981 (10.1)	46,510 (18.1)	0.23	40,747 (15.9)	50,484 (17.1)	0.03
Thiazolidinediones	1,937 (4.9)	20,254 (7.9)	0.12	17,607 (6.9)	21,979 (7.4)	0.02
No. of drugs for diabetes mellitus, average (SD)	0.82 (1.1)	0.86 (0.9)	0.05	0.85 (1.0)	0.86 (1.0)	<0.01
No. of drugs for diabetes mellitus						
0	20,777 (52.9)	112,608 (43.9)	0.18	122,409 (47.6)	131,776 (44.5)	0.06
1	9,601 (24.5)	84,258 (32.9)	0.19	72,838 (28.3)	96,529 (32.6)	0.09
2	5,308 (13.5)	43,344 (16.9)	0.09	42,848 (16.7)	48,905 (16.5)	<0.01
>2	3,565 (9.1)	16,088 (6.3)	0.11	18,888 (7.3)	18,643 (6.3)	0.04

Characteristics	Before IPTW			After IPTW		
	SGLT2i patients (n=39,251)	DPP4i patients (n=256,298)	SMD	SGLT2i patients (weighted n=256,983)	DPP4i patients (weighted n=295,853)	SMD
Diuretics						
Loop diuretics	5,908 (15.1)	20,547 (8.0)	0.22	22,262 (8.7)	26,603 (9.0)	0.01
Potassium-sparing agents	4,118 (10.5)	9,503 (3.7)	0.27	11,782 (4.6)	13,732 (4.6)	<0.01
Thiazide-like diuretics	495 (1.3)	3,116 (1.2)	0.00	3,179 (1.2)	3,594 (1.2)	<0.01
Thiazides	2,029 (5.2)	12,981 (5.1)	0.01	13,472 (5.2)	15,064 (5.1)	0.01
Vasopressin antagonists	1,316 (3.4)	1,392 (0.5)	0.20	2,630 (1.0)	2,782 (0.9)	0.01
Other drugs						
Antibacterials	15,049 (38.3)	97,387 (38.0)	0.01	98,395 (38.3)	112,781 (38.1)	<0.01
Anticoagulant agents	5627 (14.3)	18592 (7.3)	0.23	21,074 (8.2)	24,230 (8.2)	<0.01
Antivirals	623 (1.6)	4,229 (1.7)	0.01	4,272 (1.7)	4,904 (1.7)	<0.01
Beta blocking agents	9,712 (24.7)	34,565 (13.5)	0.29	39,630 (15.4)	44,308 (15.0)	0.01
Calcium channel blockers with mainly vascular effects	18,111 (46.1)	129,075 (50.4)	0.09	129,356 (50.3)	147,592 (49.9)	0.01
Corticosteroids	3,775 (9.6)	25,222 (9.8)	0.01	25,859 (10.1)	29,074 (9.8)	0.01
Histamine H2 receptor antagonists	2,677 (6.8)	22,862 (8.9)	0.08	21,441 (8.3)	25,540 (8.6)	0.01
HMG-CoA reductase inhibitors	20,548 (52.4)	119,351 (46.6)	0.12	124,686 (48.5)	139,722 (47.2)	0.03
Opioids	1,912 (4.9)	12,467 (4.9)	0.00	12,645 (4.9)	14,536 (4.9)	<0.01
Other lipid modifying agents	5,439 (13.9)	23,113 (9.0)	0.15	27,742 (10.8)	28,459 (9.6)	0.04
Platelet aggregation inhibitors	11,324 (28.9)	73,799 (28.8)	0.00	72,433 (28.2)	85,245 (28.8)	0.01
Proton pump inhibitors	13,504 (34.4)	80,889 (31.6)	0.06	81,406 (31.7)	94,895 (32.1)	0.01

Characteristics	Before IPTW					After IPTW				
	SGLT2i patients (n=39,251)		DPP4i patients (n=256,298)		SMD	SGLT2i patients (weighted n=256,983)		DPP4i patients (weighted n=295,853)		SMD
RASIs	21,966	(56.0)	123,979	(48.4)	0.15	127,251	(49.5)	145,513	(49.2)	0.01
X-ray contrast media, Iodinated	754	(1.9)	5,601	(2.2)	0.02	5,316	(2.1)	6,436	(2.2)	0.01
Parameters of specific health checkups										
Received in the prior year, n (%)	8,896	(22.7)	48,090	(18.8)	0.10	53,651	(20.9)	57,154	(19.3)	0.04
BMI										
No. of patients with measurement, n (%)	8,880	(22.6)	47,859	(18.7)	0.10	53,573	(20.8)	56,909	(19.2)	0.04
BMI, kg/m², average (SD)	26.6	(4.7)	24.5	(4.2)	0.55	25.4	(1.9)	24.8	(1.9)	0.14
≥30	1,808	(20.4)	3,816	(8.0)	0.36	6,137	(11.5)	5,788	(10.2)	0.04
<30 and ≥25	3,590	(40.4)	15,578	(32.5)	0.16	19,487	(36.4)	19,218	(33.8)	0.06
<25 and ≥18.5	3,360	(37.8)	26,800	(56.0)	0.37	26,685	(49.8)	30,116	(52.9)	0.06
<18.5	122	(1.4)	1,665	(3.5)	0.14	1,264	(2.4)	1,787	(3.1)	0.05
HbA1c										
No. of patients with measurement, n (%)	8,599	(21.9)	46,679	(18.2)	0.09	52,076	(20.3)	55,428	(18.7)	0.04
HbA1c, %, average (SD)	7.0	(1.7)	6.9	(1.6)	0.09	7.1	(0.5)	7.0	(0.5)	0.08
≥8.0	1,320	(15.4)	4,843	(10.4)	0.15	7,075	(13.6)	6,335	(11.4)	0.04
<8.0 and ≥7.0	2,304	(26.8)	13,031	(27.9)	0.03	15,233	(29.3)	15,347	(27.7)	0.03
<7.0 and ≥6.0	3,915	(45.5)	24,960	(53.5)	0.16	25,676	(49.3)	28,901	(52.1)	0.01
<6.0	1,060	(12.3)	3,845	(8.2)	0.14	4,093	(7.9)	4,845	(8.7)	<0.01
Systolic blood pressure										

Characteristics	Before IPTW					After IPTW				
	SGLT2i patients (n=39,251)		DPP4i patients (n=256,298)		SMD	SGLT2i patients (weighted n=256,983)		DPP4i patients (weighted n=295,853)		SMD
No. of patients with measurement, n (%)	8,886	(22.6)	47,877	(18.7)	0.10	53,597	(20.9)	56,931	(19.2)	0.04
Systolic blood pressure, mmHg, average (SD)	130	(17.1)	133	(18.7)	0.13	132	(7.6)	132	(7.6)	0.04
≥140	2,302	(25.9)	14,721	(30.7)	0.11	16,067	(30)	17,114	(30.1)	0.02
<140	6,584	(74.1)	33,156	(69.3)	0.11	37,530	(70)	39,818	(69.9)	0.03
Diastolic blood pressure										
No. of patients with measurement, n (%)	8,886	(22.6)	47,877	(18.7)	0.10	53,597	(20.9)	56,931	(19.2)	0.04
Diastolic blood pressure, mmHg, average (SD)	76	(11.4)	74	(11.9)	0.16	75	(4.93)	75	(4.93)	0.06
≥90	923	(10.4)	3,889	(8.1)	0.08	4,959	(9.3)	4,837	(8.5)	0.02
<90	7,963	(89.6)	43,988	(91.9)	0.08	48,638	(90.7)	52,095	(91.5)	0.03
Urine glucose (Qualitative test) [a]										
No. of patients with measurement, n (%)	8,800	(22.4)	47,450	(18.5)	0.10	53,120	(20.7)	56,433	(19.1)	0.04
−	2,550	(29.0)	38,250	(80.6)	1.21	14,451	(27.2)	45,213	(80.1)	1.25
±	158	(1.8)	2,095	(4.4)	0.15	1,033	(1.9)	2,488	(4.4)	0.14
1+	504	(5.7)	2,574	(5.4)	0.01	3,219	(6.1)	3,042	(5.4)	0.03
2+	1,042	(11.8)	1,971	(4.2)	0.29	7,074	(13.3)	2,415	(4.3)	0.32
3+	4,546	(51.7)	2,560	(5.4)	1.19	27,343	(51.5)	3,275	(5.8)	1.17
Urine protein (Qualitative test)										
No. of patients with measurement, n (%)	8,810	(22.4)	47,458	(18.5)	0.10	53,177	(20.7)	56,442	(19.1)	0.04
−	6,511	(73.9)	35,344	(74.5)	0.01	39,123	(73.6)	41,928	(74.3)	0.02

Characteristics	Before IPTW					After IPTW				
	SGLT2i patients (n=39,251)		DPP4i patients (n=256,298)		SMD	SGLT2i patients (weighted n=256,983)		DPP4i patients (weighted n=295,853)		SMD
±	1,093	(12.4)	5,957	(12.6)	0.00	7,044	(13.2)	7,169	(12.7)	0.02
1+	717	(8.1)	3,916	(8.3)	0.00	4,324	(8.1)	4,638	(8.2)	<0.01
2+	345	(3.9)	1,626	(3.4)	0.03	1,893	(3.6)	1,953	(3.5)	0.01
3+	144	(1.6)	615	(1.3)	0.03	793	(1.5)	753	(1.3)	0.01
Serum creatinine[a]										
No. of patients with measurement, n (%)	7,387	(18.8)	41,590	(16.2)	0.07	46,281	(18.0)	49,137	(16.6)	0.04
Serum creatinine, mg/dL, average (SD)	0.85	(0.4)	0.82	(0.4)	0.11	0.82	(0.1)	0.82	(0.1)	0.02
eGFR										
No. of patients with measurement, n (%)	7,387	(18.8)	41,590	(16.2)	0.07	46,281	(18.0)	49,137	(16.6)	0.04
eGFR, mL/min/1.73 m^2, average (SD)	68.9	(19.7)	68.2	(18.2)	0.04	69.4	(7.83)	68.3	(7.83)	0.06
≥60	5,003	(67.7)	28,529	(68.6)	0.02	32,410	(70.0)	33,724	(68.6)	0.03
<60 and ≥30	2,234	(30.2)	12,506	(30.1)	0.00	13,292	(28.7)	14,721	(30.0)	0.03
<30	150	(2.0)	555	(1.3)	0.05	579	(1.3)	693	(1.4)	0.01

[a]Parameters not used to calculate propensity scores. Insulin and glucagon-like peptide-1 analogs were defined as those used within the past 90 days, and iodinated X-ray contrast media were defined as those used within the past 3 days. Parameters of specific health checkups used only categorized data to calculate the propensity scores. Urinary glucose and protein were assessed as part of the Japanese nationwide specific health checkup program, using dipstick test results reported on a semi-quantitative scale (−, ±, 1+, 2+, 3+).

AKI, acute kidney injury; BMI, body mass index; CKD, chronic kidney disease; DPP4i, dipeptidyl peptidase-4 inhibitor; IPTW, inverse probability of treatment weighting; eGFR, estimated glomerular filtration rate; HbA1c, hemoglobin A1c; NSAID, nonsteroidal anti-inflammatory drug; RASIs, renin–

angiotensin system inhibitors; SD, standard deviation; SGLT2i, sodium–glucose cotransporter-2 inhibitor; SMD, standardized mean difference; T2DM, type 2 diabetes mellitus.

Supplementary Table S6 Baseline characteristics of the SGLT2i and DPP4i cohorts after PSM

Characteristics	After PSM				
	SGLT2i patients (n=33,839)		DPP4i patients (n=33,839)		SMD
Male, n (%)	19,643	(58.0)	19,921	(58.9)	0.02
Age, years, average (SD)	71.5	(12.5)	71.1	(12.2)	0.03
Calendar year on index date, n (%)					
2015	36	(0.1)	27	(0.1)	0.01
2016	570	(1.7)	589	(1.7)	<0.01
2017	1,330	(3.9)	1,362	(4.0)	0.01
2018	2,181	(6.4)	2,165	(6.4)	<0.01
2019	3,962	(11.7)	3,978	(11.8)	<0.01
2020	4,791	(14.2)	4,735	(14.0)	0.01
2021	7,219	(21.3)	7,198	(21.3)	<0.01
2022	10,610	(31.4)	10,634	(31.4)	<0.01
2023	3,140	(9.3)	3,151	(9.3)	<0.01
Hospitalization on index date, n (%)	2,949	(8.7)	2,875	(8.5)	0.01
Surgical procedure within 30 days before index date, n (%)	3,949	(11.7)	3,946	(11.7)	<0.01
Medical history, n (%)					
AKI	199	(0.6)	188	(0.6)	<0.01
Angina pectoris	10,737	(31.7)	10,680	(31.6)	<0.01
Atrial fibrillation	5,479	(16.2)	5,381	(15.9)	0.01
Cancer	6,259	(18.5)	6,100	(18.0)	0.01
CKD	4,105	(12.1)	3,982	(11.8)	0.01
Dehydration	6,782	(20.0)	6,672	(19.7)	0.01
Dyslipidemia	26,810	(79.2)	26,777	(79.1)	<0.01
Heart failure	13,405	(39.6)	13,170	(38.9)	0.01
Hypertension	27,857	(82.3)	27,851	(82.3)	<0.01
Liver diseases	13,203	(39.0)	13,259	(39.2)	<0.01
Myocardial infarction	2,112	(6.2)	2,074	(6.1)	0.01
Non-diabetic acidosis	439	(1.3)	429	(1.3)	<0.01
Obesity	1,007	(3.0)	1,012	(3.0)	<0.01
Other renal disease	6,731	(19.9)	6,638	(19.6)	0.01
Peripheral vascular disease	9,121	(27.0)	9,110	(26.9)	<0.01
Rheumatoid arthritis	1,472	(4.4)	1,472	(4.4)	<0.01
Stroke	5,301	(15.7)	5,175	(15.3)	0.01

Characteristics	After PSM		
	SGLT2i patients (n=33,839)	DPP4i patients (n=33,839)	SMD
T2DM with ketoacidosis	174 (0.5)	161 (0.5)	0.01
T2DM with neurological complications	2,376 (7.0)	2,397 (7.1)	<0.01
T2DM with ophthalmic complications	8,590 (25.4)	8,630 (25.5)	<0.01
T2DM with peripheral circulatory complications	277 (0.8)	279 (0.8)	<0.01
T2DM with renal complications	6,105 (18.0)	6,094 (18.0)	<0.01
Venous thromboembolism	1,936 (5.7)	1,907 (5.6)	<0.01
Medication, n (%)			
Drugs for diabetes mellitus			
Alpha glucosidase inhibitors	2,767 (8.2)	2,793 (8.3)	<0.01
Biguanides	8,647 (25.6)	8,799 (26.0)	0.01
Glinides	1,457 (4.3)	1,449 (4.3)	<0.01
Glucagon-like peptide-1 analogs[a]	579 (1.7)	455 (1.3)	0.03
Imeglimin	44 (0.1)	49 (0.1)	<0.01
Insulins and analogs	3,560 (10.5)	3,607 (10.7)	0.01
Sulfonylureas	3,229 (9.5)	3,317 (9.8)	0.01
Thiazolidinediones	1,533 (4.5)	1,563 (4.6)	<0.01
No. of drugs for diabetes mellitus, average (SD)	0.64 (1.0)	0.65 (1.0)	0.01
No. of drugs for diabetes mellitus			
0	19,562 (57.8)	18,185 (53.7)	0.08
1	8,787 (26.0)	10,635 (31.4)	0.12
2	3,876 (11.5)	3,857 (11.4)	0.00
>2	1,614 (4.8)	1,162 (3.4)	0.07
Diuretics			
Loop diuretics	4,754 (14.0)	4,625 (13.7)	0.01
Potassium-sparing agents	3,147 (9.3)	3,016 (8.9)	0.01
Thiazide-like diuretics	421 (1.2)	446 (1.3)	0.01
Thiazides	1,753 (5.2)	1,797 (5.3)	0.01
Vasopressin antagonists	846 (2.5)	798 (2.4)	0.01
Other drugs			
Antibacterials	12,937 (38.2)	12,961 (38.3)	<0.01
Anticoagulant agents	4,628 (13.7)	4,522 (13.4)	0.01
Antivirals	540 (1.6)	563 (1.7)	0.01
Beta blocking agents	7,955 (23.5)	7,895 (23.3)	<0.01

Characteristics	After PSM		
	SGLT2i patients (n=33,839)	DPP4i patients (n=33,839)	SMD
Calcium channel blockers with mainly vascular effects	15,952 (47.1)	16,154 (47.7)	0.01
Corticosteroids	3,306 (9.8)	3,329 (9.8)	<0.01
Histamine H2 receptor antagonists	2,365 (7.0)	2,280 (6.7)	0.01
HMG-CoA reductase inhibitors	17,313 (51.2)	17,313 (51.2)	<0.01
Opioids	1,672 (4.9)	1,698 (5.0)	<0.01
Other lipid modifying agents	4,423 (13.1)	4,542 (13.4)	0.01
Platelet aggregation inhibitors	9,549 (28.2)	9,534 (28.2)	<0.01
Proton pump inhibitors	11,468 (33.9)	11,446 (33.8)	<0.01
RASIs	18,632 (55.1)	18,537 (54.8)	0.01
X-ray contrast media, Iodinated	651 (1.9)	671 (2.0)	<0.01
Parameters of specific health checkups			
Received in the prior year, n (%)	7,761 (22.9)	7,845 (23.2)	0.01
BMI			
No. of patients with measurement, n (%)	7,746 (22.9)	7,831 (23.1)	0.01
Body mass index, kg/m², average (SD)	26.4 (4.49)	26.2 (4.5)	0.03
≥30	1,419 (18.3)	1,474 (18.8)	0.01
<30 and ≥25	3,147 (40.6)	3,241 (41.4)	0.02
<25 and ≥18.5	3,072 (39.7)	3,006 (38.4)	0.03
<18.5	108 (1.4)	110 (1.4)	<0.01
HbA1c			
No. of patients with measurement, n (%)	7,505 (22.2)	7,592 (22.4)	0.01
HbA1c, %, average (SD)	7.0 (1.7)	7.0 (1.8)	0.02
≥8.0	1,044 (13.9)	1,082 (14.3)	0.01
<8.0 and ≥7.0	1,965 (26.2)	2,008 (26.4)	0.01
<7.0 and ≥6.0	3,606 (48.0)	3,614 (47.6)	0.01
<6.0	890 (11.9)	888 (11.7)	0.01
Systolic blood pressure			
No. of patients with measurement, n (%)	7,751 (22.9)	7,835 (23.2)	0.01
Systolic blood pressure, mmHg, average (SD)	131 (17.2)	132 (16.9)	0.05
≥140	2,072 (26.7)	2,140 (27.3)	0.01
<140	5,679 (73.3)	5,695 (72.7)	0.01
Diastolic blood pressure			
No. of patients with measurement, n (%)	7,751 (22.9)	7,835 (23.2)	0.01

Characteristics	After PSM SGLT2i patients (n=33,839)		DPP4i patients (n=33,839)		SMD
Diastolic blood pressure, mmHg, average (SD)	76	(11.4)	76	(11.3)	<0.01
≥90	791	(10.2)	843	(10.8)	0.02
<90	6,960	(89.8)	6,992	(89.2)	0.02
Urine glucose (Qualitative test) [a]					
No. of patients with measurement, n (%)	7,675	(22.7)	7,757	(22.9)	0.01
−	2,314	(30.1)	6,137	(79.1)	1.13
±	149	(1.9)	340	(4.4)	0.14
1+	443	(5.8)	397	(5.1)	0.03
2+	921	(12.0)	359	(4.6)	0.27
3+	3,848	(50.1)	524	(6.8)	1.10
Urine protein (Qualitative test)					
No. of patients with measurement, n (%)	7,684	(22.7)	7,757	(22.9)	0.01
−	5,687	(74.0)	5,755	(74.2)	<0.01
±	971	(12.6)	1,004	(12.9)	0.01
1+	617	(8.0)	619	(8.0)	<0.01
2+	294	(3.8)	279	(3.6)	0.01
3+	115	(1.5)	100	(1.3)	0.02
Serum creatinine[a]					
No. of patients with measurement, n (%)	6,532	(19.3)	6,596	(19.5)	0.01
Serum creatinine, mg/dL, average (SD)	0.84	(0.4)	0.84	(0.4)	0.01
eGFR					
No. of patients with measurement, n (%)	6,532	(19.3)	6,596	(19.5)	0.01
eGFR, mL/min/1.73 m^2, average (SD)	68.8	(19.4)	69.0	(21.8)	0.01
≥60	4,450	(68.1)	4,566	(69.2)	0.02
<60 and ≥30	1,958	(30.0)	1,929	(29.2)	0.02
<30	124	(1.9)	101	(1.5)	0.03

[a]Parameters not used to calculate propensity scores. Insulin and glucagon-like peptide-1 analogs were defined as those used within the past 90 days, and iodinated X-ray contrast media was defined as those used within the past 3 days. The parameters of specific health checkups used only categorized data to calculate the propensity scores. Urinary glucose and protein were assessed as part of the Japanese nationwide specific health checkup program, using dipstick test results reported on a semi-quantitative scale (−, ±, 1+, 2+, 3+). AKI, acute kidney injury; BMI, body mass index; CKD, chronic kidney disease; DPP4i, dipeptidyl peptidase-4 inhibitor; eGFR, estimated glomerular filtration rate; HbA1c, hemoglobin A1c; NSAID, nonsteroidal anti-inflammatory drugs; PSM, propensity score matching; RASIs, renin–angiotensin system

inhibitors; SD, standard deviation; SGLT2i, sodium–glucose cotransporter-2 inhibitor; SMD, standardized mean difference; T2DM, type 2 diabetes mellitus.

Supplementary Table S7 Primary and secondary outcomes after PSM

Outcomes	After PSM		
	SGLT2i patients (n=33,839)	DPP4i patients (n=33,839)	RR or IRR [95% CI]
AKI within 90 days of NSAID initiation, n, (%)	59 (0.17)	88 (0.26)	0.67 [0.48-0.93]
AKI within 30 days of NSAID initiation, n, (%)	49 (0.14)	76 (0.22)	0.64 [0.45-0.92]
AKI incidence during the period of NSAID co-administration (up to 90 days), n, (%)	22 (0.07)	34 (0.10)	−
Duration during the period of NSAID co-administration (up to 90 days), days	646,527	699,608	−
AKI incidence during the period of NSAID co-administration (up to 90 days), 1,000 person-years, [95% CI]	12.4 [7.34-18.07]	17.7 [12.0-24.0]	0.70 [0.41-1.20]

AKI, acute kidney injury; CI, confidence interval; IRR, incidence rate ratio; NSAID, nonsteroidal anti-inflammatory drugs; PSM, propensity score matching; RR, risk ratio.

Supplementary Table S8 Primary and secondary outcomes for the expanded outcome definition after IPTW

Outcomes	Defining AKI as either ICD-10 code N17X or N10 diagnosis			Defining AKI as either ICD-10 code N17X diagnosis or initiation of dialysis		
	SGLT2i patients (n=255,302)	DPP4i patients (n=293,719)	RR or IRR [95% CI]	SGLT2i patients (n=256,984)	DPP4i patients (n=295,853)	RR or IRR [95% CI]
AKI within 90 days of NSAID initiation, weighted n, (%)	910 (0.36)	1,787 (0.61)	0.70 [0.65-0.76]	562 (0.22)	1,003 (0.34)	0.65 [0.58-0.72]
AKI within 30 days of NSAID initiation, weighted n, (%)	439 (0.17)	752 (0.26)	0.67 [0.60-0.76]	259 (0.10)	515 (0.17)	0.58 [0.50-0.67]
AKI incidence during the period of NSAID co-administration (up to 90 days), weighted n, (%)	440 (0.17)	633 (0.22)	–	238 (0.09)	414 (0.14)	–
Duration during the period of NSAID co-administration (up to 90 days), days	4,940,172	6,125,067	–	4,969,823	6,165,633	–
AKI incidence during the period of NSAID co-administration (up to 90 days), 1,000 person-years, [95% CI]	32.5 [29.5-35.6]	37.7 [34.8-40.7]	0.86 [0.76-0.97]	17.4 [15.3−19.8]	24.5 [22.2−26.9]	0.71 [0.61-0.84]

AKI, acute kidney injury; CI, confidence interval; ICD-10, International Classification of Diseases, 10th edition; IRR, incidence rate ratio; NSAID, nonsteroidal anti-inflammatory drugs; RR, risk ratio.
